# Myogenic Potential of Canine Craniofacial Satellite Cells

**DOI:** 10.3389/fnagi.2014.00090

**Published:** 2014-05-13

**Authors:** Rita Maria Laura La Rovere, Mattia Quattrocelli, Tiziana Pietrangelo, Ester Sara Di Filippo, Lisa Maccatrozzo, Marco Cassano, Francesco Mascarello, Inès Barthélémy, Stephane Blot, Maurilio Sampaolesi, Stefania Fulle

**Affiliations:** ^1^Department of Neuroscience and Imaging, University “G. d’Annunzio” Chieti-Pescara, Chieti, Italy; ^2^Interuniversity Institute of Myology (IIM), University “G. d’Annunzio” Chieti-Pescara, Chieti, Italy; ^3^Center for Excellence on Ageing (CeSI), G d’Annunzio Foundation, Chieti, Italy; ^4^Translational Cardiomyology Laboratory, Stem Cell Biology and Embryology, Department of Development and Regeneration, KU Leuven, Leuven, Belgium; ^5^Department of Experimental Veterinary Sciences, Faculty of Veterinary Medicine, University of Padua, Padua, Italy; ^6^School of Life Sciences, Ecole Polytechnique Fédérale de Lausanne, Lausanne, Switzerland; ^7^Department of Comparative Biomedicine and Food Safety, University of Padua, Padua, Italy; ^8^Laboratoire de Neurobiologie, Ecole Nationale Vétérinaire d’Alfort, Maisons-Alfort, France; ^9^Department of Public Health, Experimental and Forensic Medicine, Division of Human Anatomy, University of Pavia, Pavia, Italy

**Keywords:** presomitic and somitic satellite cells, differentiation, dystrophic muscle, microRNA

## Abstract

The skeletal fibers have different embryological origin; the extraocular and jaw-closer muscles develop from prechordal mesoderm while the limb and trunk muscles from somites. These different origins characterize also the adult muscle stem cells, known as satellite cells (SCs) and responsible for the fiber growth and regeneration. The physiological properties of presomitic SCs and their epigenetics are poorly studied despite their peculiar characteristics to preserve muscle integrity during chronic muscle degeneration. Here, we isolated SCs from canine somitic [somite-derived muscle (SDM): vastus lateralis, rectus abdominis, gluteus superficialis, biceps femoris, psoas] and presomitic [pre-somite-derived muscle (PSDM): lateral rectus, temporalis, and retractor bulbi] muscles as myogenic progenitor cells from young and old animals. In addition, SDM and PSDM-SCs were obtained also from golden retrievers affected by muscular dystrophy (GRMD). We characterized the lifespan, the myogenic potential and functions, and oxidative stress of both somitic and presomitic SCs with the aim to reveal differences with aging and between healthy and dystrophic animals. The different proliferation rate was consistent with higher telomerase activity in PSDM-SCs compared to SDM-SCs, although restricted at early passages. SDM-SCs express early (Pax7, MyoD) and late (myosin heavy chain, myogenin) myogenic markers differently from PSDM-SCs resulting in a more efficient and faster cell differentiation. Taken together, our results showed that PSDM-SCs elicit a stronger stem cell phenotype compared to SDM ones. Finally, myomiR expression profile reveals a unique epigenetic signature in GRMD SCs and miR-206, highly expressed in dystrophic SCs, seems to play a critical role in muscle degeneration. Thus, miR-206 could represent a potential target for novel therapeutic approaches.

## Introduction

Satellite cells (SCs) are quiescent adult stem cells and they are located between the basal lamina and sarcolemma of myofibers (Mauro, 1961). Since SCs function to repair skeletal muscle damaged by injury or disease, they are responsible for muscle preservation and growth.

Satellite cells exhibit limited gene expression and protein synthesis due to extremely low cellular turnover (Rando, 2006) and upon activation they leave the quiescent state and move outside of the basal lamina, enter the cell cycle and proliferate. The proliferating SCs, known as myogenic progenitor cells, co-express Pax7 and MyoD, and undergo multiple rounds of division (Chargé and Rudnicki, 2004). Subsequently, SCs withdraw from the cell cycle down-regulating Pax7 and expressing myogenin in order to differentiate into multinucleated myofibers. A limited number of SCs maintain Pax7 for self-renewing and enter again a quiescent state. The fact that activated SCs can loose the expression of myogenic markers or eventually leave the cell cycle shows that they are a heterogeneous mixture of stem cells and committed progenitors. The myogenic commitment of SCs is finely regulated by the expression of a temporal class of myogenic transcription factors (MRFs), which are important for the renewal of SCs (Porter et al., 2006; McLoon et al., 2007) or for determining their differentiation. However, the signaling pathways that maintain or suppress SCs at different functional states are largely unknown and recent studies have emphasized the role of microRNAs (miRNAs) as novel post-transcriptional modulators of myogenic gene expression (Drummond et al., 2010). miRNAs are small non-coding RNAs that negatively regulate gene expression by binding mRNA target and interfering with protein synthesis, also by reducing the accumulation of target messengers. A recent analysis found that between 74 and 92% of the transcriptome is potentially regulated by miRNAs (Miranda et al., 2006).

Emerging evidence has demonstrated that miRNAs are essential for several biological functions including homeostasis, apoptosis (Huang et al., 2012), and deleterious changes in miRNA expression are associated with human diseases (Chen et al., 2008). Several muscle-specific miRNAs in heart and skeletal muscle (myomiRs) including miR-1, miR-26a, miR-125b, miR-133, miR-206, miR-208, etc. (McCarthy and Esser, 2007; Small et al., 2010) are considered important for myoblast differentiation, proliferation, and muscle remodeling. Among the various myomiRs, miR-1 and miR-133 are involved in the modulation of muscle growth and differentiation, while miR-206 specifically promotes muscle myogenesis. The regulation of these myomiRs is controlled by MRFs, as MyoD and myogenin (Rao et al., 2006). SC regenerative potential is impaired in Duchenne muscular dystrophies (DMD), a recessive X-linked form of muscular degeneration, and in age-related loss of muscle mass, i.e., a condition known as sarcopenia (Snijders et al., 2009). In DMD patients, the SC-mediated regenerative process is not sufficient to rescue the phenotype, resulting in a general tissue inflammation. This culminates in continuous cycles of degeneration/regeneration that finally deplete the pool of skeletal muscle stem cells. However, masseter (Mas), extraocular, laryngeal, and pharyngeal muscles are less affected during sarcopenic and dystrophic degeneration. These muscles present peculiar isoforms of myosin heavy chain (MyHC) proteins, as MyHC 2B, EO, alpha-cardiac, and M. For this peculiarity, some authors refer to cranial facial muscles as “special muscles” (Sciote et al., 1994; Toniolo et al., 2008; Schiaffino and Reggiani, 2011). The embryonic origin of these special muscles is the presomitic mesoderm while trunk, leg, and arm muscles derive from somites. As described by Biressi and Rando (2010), signaling pathways responsible for myogenic differentiation of SCs isolated from trunk and special muscles are different. Interestingly, eye extrinsic muscles responsible for complex and highly coordinated movements do not show signs of sarcopenia and are not affected by DMD (McLoon et al., 2007).

The muscular dystrophy golden retriever (GRMD) dog is the closest pathological counterpart of DMD patients (Collins and Morgan, 2003). In addition, sarcopenia is an emerging syndrome of importance in dogs (Freeman, 2012). Here, we report for the first time the characterization of SCs isolated from presomitic muscles of young, old, and GRMD dogs. We focus on stem cell properties during aging and in the dystrophic context, highlighting the epigenetic signature of SC-myotube transition.

## Materials and Methods

### Sampling of canine biopsies

Muscle biopsies were collected from different institutions involved in this study: (1) Veterinary University Clinics in Padua; (2) Veterinary School of Maison-Alfort, Paris. In Padua, the samples were obtained from dogs that had been euthanized in the Veterinary University Clinics. Those dogs were diagnosed as terminally ill following accidents. The muscle tissues were collected during the normal surgical procedures. Biopsies from GRMD were obtained from the Veterinary School of Maison-Alfort (Paris, France). All GRMD dogs considered in this study (see Table 1) were maintained until natural death, and underwent necropsy only upon decease. The muscle sampling was done according to Institutional Animal Care and Use Committee recommendations and according to the local ethical rules and the Declaration of Helsinki. The muscles were divided according to their embryonic origin, MyHC isoform composition, and the anatomical location (Table 1). Trunk and limb muscles: *vastus lateralis* (VL), *psoas, rectus abdominis* (RA), *gluteus superficialis* (GS), *biceps femoris* (BF) as somite-derived muscles (SDM). Head muscles: *rectus lateralis* (RL), *retractor bulbi* (RB), *temporalis-M fiber* (MT), *Mas* as pre-somite-derived muscle (PSDM). GRMD SCs were collected only from young dog, while samples from wild-type (WT) dogs were collected from dogs of different ages.

**Table 1:** **Summary of canine muscle biopsies**.

Group	Dog	Muscle biopsies	Age	Embryonic origin
**HEALTHY YOUNG DOGS**				
	#1	*Rectus abdominis*	1 year	Somites
	#2	*Gluteus superficialis*	3 years	Somites
		*Rectus abdominis*		Somites
	#3	*Rectus abdominis*	10 months	Somites
	#4	*Biceps femoris*	8 months	Somites
	#5	*Extraocular*	1 year	Presomitic cranial mesoderm
		*Vastus lateralis*		Somites
	#6	*Extraocular*	5 months	Presomitic cranial mesoderm
		*Vastus lateralis*		Somites
	#7	*Extraocular*	1 year	Presomitic cranial mesoderm
		*Vastus lateralis*		Somites
	#8	*Rectus abdominis*	1 year	Somites
		*Vastus lateralis*		Somites
**HEALTHY AGED DOGS**				
	#9	*Psoas*	9 years	Presomitic cranial mesoderm
		*Temporalis, M fiber*		Presomitic cranial mesoderm
		*Rectus lateralis*		Presomitic cranial mesoderm
	#10	*Masseter*	18 years	Presomitic cranial mesoderm
		*Rectus lateralis*		Presomitic cranial mesoderm
		*Retractor bulbi*		Presomitic cranial mesoderm
	#11	*Rectus abdominis*	13 years	Somites
		*Vastus lateralis*		Somites
**GRMD**				
	#12	*Extraocular*	1 year	Presomitic cranial mesoderm
		*Vastus lateralis*		Somites
	#13	*Extraocular*	1 year	Presomitic cranial mesoderm
		*Vastus lateralis*		Somites

### Isolation and culture of canine SCs

Satellite cells were isolated from muscle tissues stored in liquid nitrogen in FBS (# ECS0100187, Euroclone, Milan, Italy) + 10% of DMSO (#D5879, Sigma-Aldrich, Milan, Italy). Frozen dissected muscle biopsies were thawed at 37°C and washed with PBS. Under sterile condition, few drops of enzymatic solution of 0.4 mg/ml collagenase type V (#C9263, Sigma-Aldrich, Milan, Italy), 0.6 mg/ml pancreatin (#P3292, Sigma-Aldrich, Milan, Italy) dissolved in PBS (# ECB4004L, Euroclone, Milan, Italy), filtered using a sterile syringe filter of 0.2 μm pore size, stored at 4°C until use. The solution was added and the muscles were minced to fine slurry using a scalpel. After transferring the minced tissue into 50 ml tubes, 10 ml of enzymatic solution was added and incubated at 37°C in a shaking incubator for 30 min. After gravity sedimentation, the supernatant was collected into new falcon tube, through filtration by 100 μm cell strainer; the digestion was stopped by adding an equal volume of filtered FBS. The cell suspension was centrifuged at 1200 rpm for 5 min, the supernatant was discarded and the cells were resuspended in growth medium (GM). GM contains (% vol/vol): DMEM high glucose (# ECB7501L, Euroclone, Milan, Italy), 0.1 gentamicin (# ECM0011B, Euroclone, Milan, Italy), 20 FBS, 1 MEM NEAA 100× (#11140, GIBCO, Life Technology, Carlsbad, CA, USA), 1 sodium pyruvate 100 mM (#11360, GIBCO, Life Technology, Carlsbad, CA, USA), 1 penicillin/streptomycin 100× (# ECB3001D, Euroclone, Milan, Italy), 1 l-glutamine 100× (#25030-024, GIBCO, Life Technology, Carlsbad, CA, USA), 2 chicken embryo extract (#2850145, MP, Santa Ana, CA, USA), 2 heat-inactivated HS (# ECS0091L, Euroclone, Milan, Italy) (56°C, 36 min) plus 5 ng/ml bFGF (# PHG0026, GIBCO, Life Technology, Carlsbad, CA, USA) in PBS. Canine cells were pre-plated for 1 h on uncoated petri dishes to permit the attachment of fibroblasts. Unattached cells were transferred to collagen I-coated 60 mm cell culture dish. Five days later, small round and poorly adhering cells appeared on collagen coated-plates and were allowed to proliferate in GM until reaching 70–80% confluence. The cells were either cryoconserved or expanded for further analysis. To generate batches, 70–80% confluent SCs were frozen for long-term storage at density of 10^6^ cells/ml in liquid nitrogen. To expand the culture, SCs were plated again until reaching 80% confluence and then again detached (passage). The population doubling level (PDL) was calculated at each passage with the following equation: log10(*N*/*n*)/ln2 with *N* as the number of cells at the time of the passage and *n* as the number of cells initially plated. At first passage, all SC populations were considered to be as 1 PDL and we evaluated the maximal PDL reached by the cultures.

### SC proliferation analysis by Edu (5-ethynyl-2′-deoxyuridine) flow cytometry

Cells were plated in six well at density of 5 × 10^4^/well; after 24 h the cells were treated with 100 μM H_2_O_2_ (Sigma). The flow cytometry method was performed at 24 and 48 h after treatment with hydrogen peroxide. Briefly, 10 μM Edu was added to the culture medium for 2 h; after the cells were harvested and washed twice with 1% BSA in PBS. The cells were fixed with 2% paraformaldehyde in PBS for 15 min at RT. After wash, the cell pellet was resuspended with permeabilization solution and incubated with FxCycle Violet for DNA stain. After an incubation period of 15 min at 37°C in the dark, cells were transferred on ice until analysis with FACS Calibur™ (BD, USA).

### Cell differentiation

Skeletal muscle differentiation was induced by culturing 5,000 cells/cm^2^ in differentiative medium (DM) for 7 days in the same medium. DM contains (% vol/vol): DMEM high glucose, 0.1 gentamicin, 5 heat-inactivated HS (56°C, 36 min), 10 μg/ml insulin, 100 μg/ml apo-transferrin (#T-2036, Sigma-Aldrich, Milan, Italy), 1 MEM NEAA 100×, 1 sodium pyruvate 100 mM, 1 penicillin/streptomycin 100×, 1 l-glutamine 100×. The fusion index (FI) was calculated as the ratio of the number of nuclei inside myotubes to the number of total nuclei in 10–20 fields for each sample. We considered myotubes exclusively the cells positive for MyHC with two or more nuclei.

### Telomerase activity

Telomerase activity was assessed by TraPEZE RT Telomerase Detection Kit (#S7710, Millipore, Merck KGaA, Darmstadt, Germany), according to the recommendations of the manufacturer. Briefly, cell pellets of the samples were resuspended in CHAPS lysis buffer and protein concentrations were measured by the Bradford Assay (#500-0205, Bio-Rad, Bio-Rad Laboratories s.r.l., Segrate, Milan, Italy). The quantification of telomerase activity was obtained from the standard curve of TRS8 template. TRS8 template is an oligonucleotide with a sequence identical to the oligonucleotide substrate primer extended with eight telomeric repeats AG(GGTTAG). The kit provided cell pellets as positive controls. According to the manufacturer’s instructions, we also performed a negative control for each sample. Negative controls were obtained incubating samples at 85°C for 10 min prior to the TRAP assay to inactivate telomerase. For each sample, 100 ng of total proteins were adjusted to a volume of 2 μl by CHAPS lysis buffer and used for the telomerase activity assay. The quantitative real-time polymerase chain reactions were performed in 96-well optical reaction plates (Applied BioSystems, Life Technologies, Molecular Device, Sunnyvale, CA, USA) using the ABI PRISM 7700 Sequence Detection System. Reactions were carried out in triplicates using the recommended Titanium Taq Polymerase (BD Clontech, #639208). The experiments were performed according to the manufacturer’s instructions. We tested telomerase activity in SC samples collected at early (II–III) and late (VIII–X) passages and expressed as log copy number calculated using threshold cycle values and the standard curve for each sample.

### Immunofluorescence assay

Satellite cells were fixed for immunocytochemistry and immunofluorescence analysis to reveal myogenic markers (MF-20 for MyHC and Pax7 Abs DSHB, USA; MyoD Abs, Dako, USA) and Ki67 (Dako, USA). Cells were washed twice with PBS and incubated with 2% PFA (#P6148, Sigma-Aldrich, Milan, Italy) for 10 min at RT. Membrane permeabilization was obtained at RT in 0.2% of Triton X-100 (#93443, Sigma-Aldrich, Milan, Italy), 1% BSA (#A9647, Sigma-Aldrich, Milan, Italy) in PBS and non-specific binding of secondary antibodies was blocked with PBS containing 5% serum from the species in which the secondary antibody was produced. Without washing, the cells were incubated for 1 h with primary antibody diluted in 1% BSA–PBS. Then, cells were washed twice with PBS and incubated in the dark for 30 min at RT with the secondary antibody, diluted 1:500 in 1% BSA + PBS. Nuclei were stained with DAPI (#D1306, Invitrogen, Life Technologies, Molecular Device, Sunnyvale, CA, USA). Unbound antibodies were washed out with PBS and samples were mounted and analyzed with a fluorescence Nikon inverted microscope Eclipse Ti-U equipped with a Qicam Fast1354 camera using Pro-Plus software (Media Cybernetics).

### Immunocytochemistry

Immunocytochemistry to reveal desmin protein was performed using LSAB + System-AP Universal kit (#K0678, Dako, Dakocytomation, Glostrup, Denmark) according to manufacturer’s protocols.

### Isolation and quantification of microRNA

Small RNA extraction from both SDM and PSDM samples was performed using the PureLink miRNA isolation kit (#K1570-01, Invitrogen, Life Technologies, Molecular Device, Sunnyvale, CA, USA) following manufacturer’s instructions. Briefly, 10^6^ cells were resuspended in 300 μl binding buffer and 300 μl of 70% alcohol was added to the cell lysate. Cell lysate was added to a spin cartridge and centrifuged for 1 min at 12,000 × *g*. Seven hundred microliters of 100% alcohol was added to the supernatant and centrifuged in a new spin cartridge for 1 min at 12,000 × *g*. The filtrate was discarded, then 500 μl wash buffer was added and centrifuged for 1 min at 12000 × *g*. This procedure was repeated twice. Sterile RNase-free water (30 μl) was added to the spin cartridge and incubated for 1 min at RT before centrifugation at maximum speed for 1 min to elute the RNA. The RNA concentration was quantified using NanoDrop™ spectrophotometer readings of A260, A260/280, and A260/230 ratios. RNA-free nuclease water was used as blank.

### RT real-time PCR for microRNAs single assay expression

RT-stem-loop real-time PCR was performed to evaluate miRNAs relative expression. Assays for miRNA profile analysis were carried out according to Applied Biosystems protocols (TaqMan miRNA assay Kit). Briefly, RT reactions containing 20 ng of small RNA preparation, specific stem-loop primers for each miRNA, 1× buffer, dNTPs reverse transcriptase, and RNAse inhibitor were incubated in Thermocycler for 30 min at 16°C, 30 min at 42°C, 5 min at 85°C, and then held at 4°C. Then, real-time PCR for miRNA expression levels was performed using miRNA specific TaqMan probes and TaqMan universal master mix in a Eppendorf Mastercycler^®^ ep realplex system in 96-well plates, in triplicate. Expression quantification was normalized vs. *miR-16* levels. Taqman-based qPCR were conducted using miRNA-specific probes (Applied Biosystems) as: has-miR-1 (UGGAAUGUAAAGAAGUAUGUAU; #002222); has-miR-206 (UGGAAUGUAAGGAAGUGUGUGG; #000510); hsamiR-133a (UUUGGUCCCCUUCAACCAGCUG; #002246); has-miR-133b (UUUGGUCCCCUUCAACCAGCUA; #002247); has-miR-16-5p (UAGCAGCACGUAAAUAUUGGCG; #000391).

The quantitative real-time polymerase chain reactions were performed in 96-well optical reaction plates (Applied BioSystems) using the ABI PRISM 7700 Sequence Detection System. Reactions were carried out in triplicates using the recommended Titanium Taq Polymerase (#639208, BD Clontech, BD Bioscience, Clontech Laboratories Inc., San Jose, CA, USA). The relative quantification of target gene expression was evaluated using the arithmetical formula 2^−ΔΔ^*^C^*^t^, according to the comparative *C*_t_ method, which represents the amount of target, as normalized to the miR-16 endogenous control. The samples were analyzed as means of the log base-10 of the ratios, log_2_ differentiated/undifferentiated. The data were reported as Log_10_ RQ, in which upregulated miRNAs have positive values and downregulated miRNAs have negative values.

### Relative quantification of myogenic factor genes by RT real-time PCR

Total RNA from isolated canine SCs was extracted using the Trizol reagent (Invitrogen, Paisley, UK), according to the manufacturer’s protocol, from about 10^6^ cells. To assess the integrity and the amount of the RNA extracted, denaturing agarose gel electrophoresis and spectrophotometric A260/280 readings were performed. Two micrograms of total RNA was reverse transcribed into cDNA with the Superscript III kit (Invitrogen) after treatment with DNAse I (Invitrogen) to remove contaminating genomic DNA. Real-time PCR reactions were achieved using an ABI 7500 real-time PCR System (Applied Biosystems, Foster City, CA, USA) with the following conditions: 1× PowerSybrGreen Master mix (containing buffer, dNTPs, SybrGreen I dye and AmpliTaq Gold^®^DNA Polymerase), 300 nM forward and reverse primers each, 1 μl cDNA in 20 μl total volume. PCR primers for the specific target genes and for the housekeeping gene (β-actin) were designed using the Primer Express 3.0 software (Applied Biosystems). All the primer sequences (Table 2) were designed to span introns in the genomic DNA in order to minimize non-specific fluorescence signals due to contaminating genomic DNA. Relative quantifications were calculated using the ΔΔ*C*_t_ method, normalized to the reference gene (beta actin) and expressed in arbitrary units as fold change as compared to the calibrator sample (1 unit).

**Table 2:** **Primer sequences for RT real-time PCR**.

Gene	Forward sequence	Reverse sequence
Myf5	5′-CTGTCTGGTCCCGAAAGAAC-3′	5′-TGATTCGATCCAC TATGCTG-3′
Myogenin	5′-AGTGACTGCAGCTCCCACAG-3′	5′-GACGTGAGAGA GTGCAGGTT-3′
MSTN	5′-CCCGTCAAGACTCCTACAACAG-3′	5′-AATGCTCTGC CAAATACCAGT-3′
MyHC 1	5′-CACCAACCTGTCCAAGTTCC-3′	5′-CCGGGCAGAT CAAGAGAAGATA-3′
β-actin	5′-CCATCTACGAGGGGTACGCCC-3′	5′-TGCTCGAAGTCC AGGGCGACGTA-3′

### Measurement of intracellular reactive oxygen species

Cellular reactive oxygen species (ROS) were quantified by the 2,7-dichlorofluorescein diacetate (DCFH-DA, #D6883, Sigma) assay using a microplate reader (Fluorometer SPECTRAmax Gemini XS, Molecular Devices), using excitation and emission wavelengths of 480 and 530 nm, respectively and analyzed by SOFTmax Pro software, according to Menghini et al. method (Menghini et al., 2011).

### NBT assay

The NBT assay is based on the reduction of NBT (Nitro blue tetrazolium chloride, #N6639, Sigma) in formazan by $O_{2}^{-}$. Reduced formazan is then quantified at the spectrophotometer. In the presence of potentially antioxidant substances, the superoxide is detoxified (scavenger action) and decreases the amount of NBT reduced, hence lower levels of reduced formazan are detected. Cells (10^6^ cells) were detached, centrifuged 5 min at 170 × *g* and resuspended in 1 mg/ml of NBT with 1 ml 0.9% NaCl. Then, the cells were left for 3 h at 37°C (incubator), centrifuged 10 min at 100 × *g* in microfuge, resuspended in 1 ml DMSO and left for 20 min at 37°C. For the assay, cells were plated in a 96-well plate (2 × 10^5^ cell/well) and assayed at the spectrophotometer at 550 nm on a scanning multi-well reader (Microplate spectrometer SPECTRAmax 190, Molecular Devices, Sunnyvale, CA, USA) according to Sozio et al. (2013).

### Calcium imaging

Satellite cells were plated at a confluence of 6,000 cells/cm^2^ in 96-well plates (Corning, Tewksbury, MA, USA). The measurements were performed using Fura-2 AM (#F1221, Invitrogen) as Ca^2+^ indicator. Myoblasts and myotubes were loaded with 5 μM Fura-2 AM in normal external solution (NES) supplemented with 10 mg/ml of BSA for 40 min at 37°C and 5% CO_2_. NES is composed of 10 mM glucose (#454337, Carlo Erba), 140 mM NaCl (#S7653, Sigma), 2.8 mM KCl (#471177, Carlo Erba), 2 mM CaCl_2_, 2 mM MgCl_2_ (Sigma), and 10 mM HEPES (#101926, ICN, Biomedicals Inc.), pH 7.4, 290–300 mOsM. After loading, the cells were rinsed and maintained in NES for 10 min at room temperature (RT), to allow the de-esterification of the probe. Myoblasts and myotubes of similar size were selected for measurement and the region of interest (ROI) was drawn around the cells. Then, living cells were sequentially excited at 340 or 380 nm with a high-speed wavelength switcher Polychrome II (Till Photonics, Germany). Fluorescence images were collected using a 40× oil objective lens, acquired using an intensified CCD camera (Hamamatsu Photonics, Hamamatsu), stored on a PC, and analyzed off-line. The acquisition time for each fluorescence emission was 0.5 s and the background fluorescence was subtracted from the signal in the ROI. The 340 and 380 traces and 340/380-ratio were recorded and analyzed using Aquacosmos software (Hamamatsu).

### Statistical analysis

Unpaired *t*-test with Welch’s correction and ANOVA as mentioned in the figure legends, were performed using Prism5 GraphPad software (Abacus Concepts, GraphPad Software, San Diego, CA, USA). A *p*-value <0.05 was considered to indicate statistical significance.

## Results

### Canine satellite cell characterization

**Figure 1:**
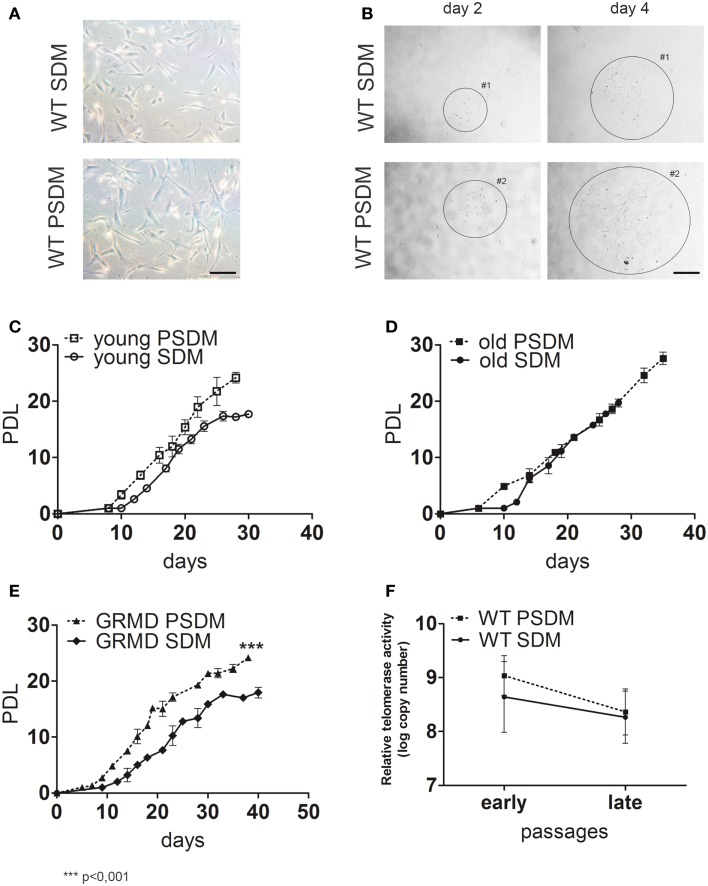
**Characterization of canine satellite cells (SCs)**. **(A,B)** Phase contrast morphology of freshly isolated SCs **(A)** from somitic (SDM) and presomitic (PSDM) muscles biopsies; bar = 50 μm. SCs were cultured at low density to generate clones **(B)** and cells were maintained in culture until they stop to divide; bar = 250 μm. Growth curves of **(C)** SDM- and PSDM-SCs from young donors; **(D)** SDM- and PSDM-SCs from old donors; **(E)** SDM and PSDM-SCs from dystrophic dogs. Telomerase activity **(F)** in SCs collected at early and late passages from SDM and PSDM. Data are represented as mean ± SD.

Following canine muscle biopsies dissociation with enzymatic solution (as described in Materials and Methods), SCs gave rise to myogenic progenitor cells in culture. To avoid confusion, we used SCs throughout the manuscript to indicate also myogenic progenitor cells, and the list of canine biopsies used to isolate SCs in our study is reported in Table 1. WT SCs isolated from somitic (SDM) and presomitic (PSDM) muscles were morphologically similar (Figure 1A) and were organized in small clones derived from single cell divisions (Figure 1B). We cultured the cells at very low density in order to identify single cells. We plated 500 cells (SDM and PSDM) on 100 mm collagen I-coated Petri dishes and marked those separate cells with a number. By counting the number of cells per clone, we evaluated the clonogenic ability of primary cell culture isolated from trunk and head muscles of young and old dogs. The ability of a single cell to proliferate independently to form a colony was similar in samples at comparable ages (data not shown).

Satellite cells in culture were expanded until replicative senescence was reached and the PDL was calculated. The growth curves of young (age ranging between 5 and 36 months) and old (age ranging between 9 and 18 years) showed higher rate of doubling level in PSDM-SCs with respect to SDM-derived SCs (Figures 1C,D). This difference was marked in SCs derived from old dogs (Figure 1D) where SDM-SCs stopped their proliferation at 25 days, after only 20 PDL. Differently, PSDM-SCs could proliferate longer (until 40 days) and undergo to senescence after about 30 PDL. No significant differences were observed between old and young SCS derived from comparable muscles. Figure 1E shows the growth curve of 1-year-old GRMD SCs isolated from SDM and PSDM samples. PSDM-SCs from GRMD samples (Figure 1E) showed a higher doublings activity in respect to SDM-SCs. This is probably due to the fact that head muscles are less affected in dystrophic dogs, and SCs were not exhausted by multiple attempts to regenerate lost tissue as occurred in more active muscles.

We tested the telomerase activity in SC samples collected at early (2, 3 p) and late (8–10 p) passages and expressed as log copy number calculated using threshold cycle values and the standard curve for each sample. As shown in Figure 1F, in PSDM-SCs there was a trend toward a higher telomerase activity (9.0 ± 0.6) compared with SDM-SCs (8.0 ± 1.0) at early passages. At late passages, the telomerase activities were similar among all analyzed samples. Immunofluorescence analysis showed that 10% of PSDM-SCs still express Ki67 after 30 days in culture, while SDM-SCs were completely negative (Figure S1 in Supplementary Material). Although we cannot exclude that SDM-SCs enter in a quiescent status, the results suggest that PSDM-SCs can still proliferate after 30 days while SDM-SCs cannot. In addition, large nuclei in SDM-SCs are further indicating senescent cells (De Cecco et al., 2011) as indicated in Figure S1 in Supplementary Material, inset and right panels.

### Expression of myogenic regulatory factors in canine SCs

**Figure 2:**
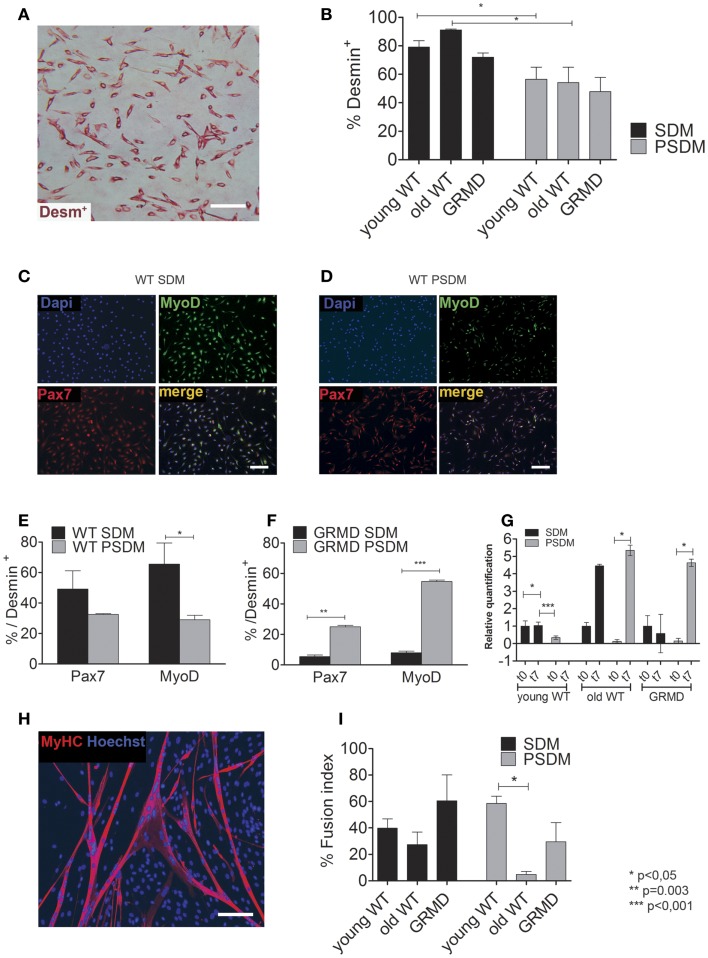
**Expression of myogenic markers in canine SCs**. **(A)** Desmin positive (Desm^+^) SCs were quantified **(B)** for WT young SCs isolated from SDM and PSDM muscle biopsies; for WT old SCs isolated from SDM and PSDM; similarly the percentage of Desm^+^ SCs in SDM and PSDM isolated from GRMD dogs. **(C,D)** Examples of immunofluorescence analysis for Pax7 and MyoD of WT SDM-SCs **(C)** and PSDM-SCs **(D)**. Quantifications of Pax7 and MyoD positive SCs **(E)** normalized to percentage of Des^+^ from WT SDM and PSDM are shown; in **(F)** are reported SCs from dystrophic dogs SDM and PSDM. Myogenin expression in SCs during differentiation was measured by quantitative real-time PCR **(G)**. Myogenin is upregulated after 7 days of differentiation in all samples expect in GRDM SDM-SCs. Three independent experiments were performed in triplicates and statistically analyzed using Dunn’s Multiple Comparison Test. MyHC staining **(H)** was used to quantify the FI **(I)** of SCs isolated from SDM and PSDM of WT young; from SDM and PSDM of WT old and of GRMD SCs isolated from SDM and PSDM. Data are represented as mean ± SD and statistically analyzed using *t*-test; *p < 0.05. Bar = 50 μm.

Taken into account the SC heterogeneity due to the adopted enzymatic digestion method, the myogenic index (MI) was estimated as a percentage of desmin (an early myogenic marker) positive (Desm^+^) cells and revealed by immunocytochemistry (Figure 2A). SCs are capable to spontaneously differentiate when they reach 80–90% of confluence, however in all experiments we differentiate SCs into myotubes by serum starvation in differentiation medium containing 2% horse serum. In general, PSDM biopsies have a lower content of myogenic cells compared to SDM samples. Age-independent differences have been found both in WT (Figure 2B, 79.1% in SDM vs. 56.5% in PSDM of young dogs, and 91.1% in SDM vs. 54.2% in PSDM of old dogs) and GRMD (72.0% in SDM vs. 47.8% in PSDM) dogs. By immunofluorescence assay (Figures 2C,D), SDM-SCs isolated from WT dog showed an higher content of Pax7^+^ (49.1 vs. 32.5%) and MyoD^+^ (65.5 vs. 29.0%) cells compared with PSDM-SCs (Figure 2E); while GRMD SCs displayed an opposite result: lower content of both Pax7^+^ (5.5 vs. 25.0%) and MyoD^+^ (8.0 vs. 54.7%) SDM vs. PSDM-SCs (Figure 2F). In addition, myogenin expression resulted upregulated during differentiation in SCs isolated from SDM and PSDM and its expression was not affected with aging (Figure 2G). However, in GRMD dogs myogenin was upregulated only in PSDM-SCs during differentiation, while SDM-SCs failed to increase the expression of myogenin at day 7 (Figure 2G).

Then, we examined the ability of SCs to fuse into multinucleated myotubes after 7 days in culture with low serum (2% HS). Figure 2H shows an example of MyHC positive canine myotubes used for the FI analysis. Young PSDM-SCs presented a higher FI compared with SDM-SCs (Figure 2I, 58.5 vs. 39.8% in PSDM vs. SDM, respectively). Interestingly, FI was drastically lower in PSDM-SCs compared to SDM-SCs isolated from old dogs (4.7 vs. 27.4%, respectively). The same trend was found in GRMD where SDM-SCs differentiate more efficiently than PSDM-SCs (60.5 vs. 29.5%). These results suggest that SCs isolated from GRMD dogs act similarly as old WT SCs.

### Oxidant levels in canine SCs

**Figure 3:**
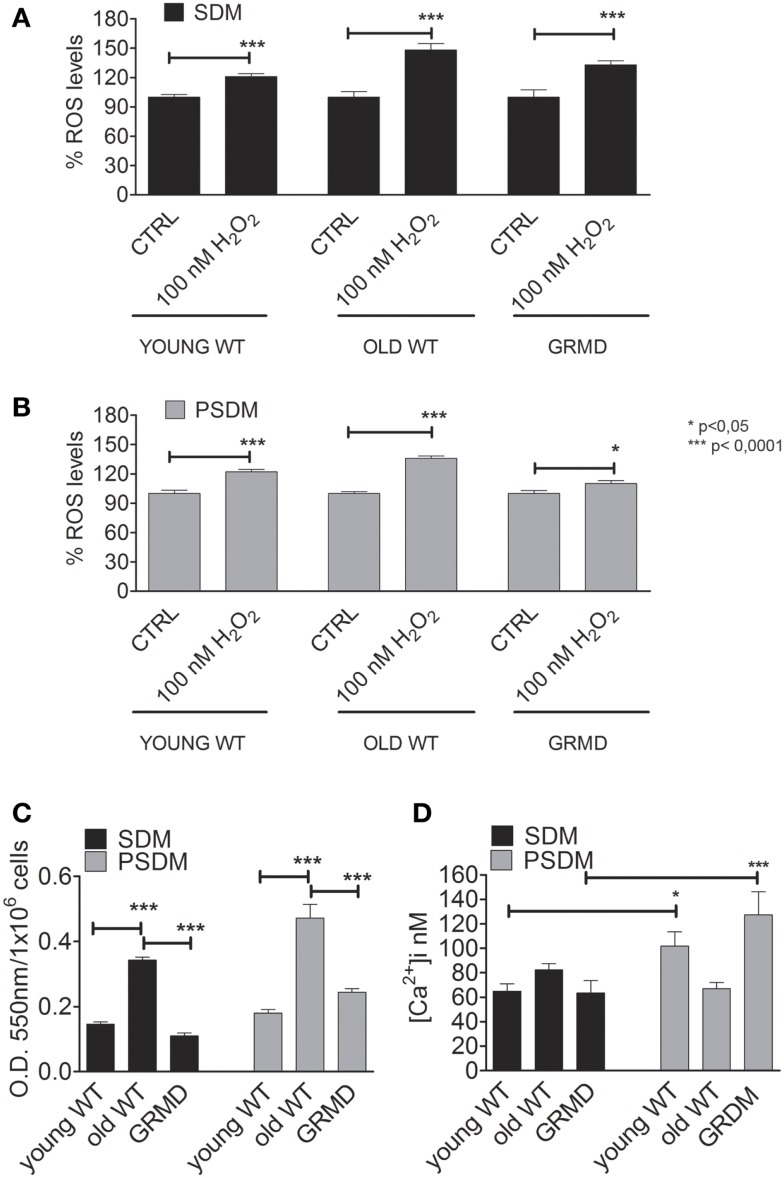
**Oxidant and calcium levels**. Quantification of intracellular ROS levels by DCFH-DA assay expressed as a percentage respect to CTRL in SCs isolated from SDM **(A,B)** PSDM. **(C)** Quantification of $O_{2}^{-}$ by rate reduction of NBT. The OD 550 nm represents the quantity of formazan revealed spectrophotometrically. **(D)** Basal levels of [Ca2^+^]_i_ expressed in nanomole measured on undifferentiated living cells by video-imaging technique. Data are represented as mean ± SD and statistically analyzed using *t*-test; *p < 0.05; **p < 0.0001.

Oxidants such as ROS/RNS are known to affect cell function, including the ability to differentiate. It has been proposed that several factors, including ROS, are able to regulate skeletal muscle gene expression. However, when the levels of oxidants consistently remain high and are not reduced by endogenous scavenger systems, as occurring in elderly skeletal muscle (Musarò et al., 2010), the cell undergoes oxidative stress, which may affect its ability to differentiate (Beccafico et al., 2007). For this reason, we analyzed the oxidant levels present in SCs isolated from muscle biopsies (Figure 3). After an oxidant insult represented by H_2_O_2_, old SCs were less capable to reduce ROS levels (SDM = 148.0 ± 26%; PSDM = 136.0 ± 9%) present in cells, demonstrating a poor antioxidant capacity (Figures 3A,B). Furthermore, the levels of superoxide anion (Figure 3C) were lower in all SDM-SCs as compared to PSDM-SCs, but in old SCs these values were very high (SDM = 0.34 ± 0.01; PSDM = 0.47 ± 0.04) as compared to young (SDM = 0.15 ± 0.01; PSDM = 0.18 ± 0.01) and GRMD (SDM = 0.11 ± 0.01; PSDM = 0.24 ± 0.01) SCs.

To address whether SCs from PSDM or SDM were still able to proliferate under stress conditions, we evaluated the effect of 100 nM H_2_O_2_ on PSDM- and SDM-SCs by Edu (5-ethynyl-2’-deoxyuridine) flow cytometry analysis (Figure S2 in Supplementary Material). The nucleoside analog EdU for thymidine substitution in cell proliferation assays has recently been proposed in FACS analysis studies (Diermeier-Daucher et al., 2009). As reported in Figure S2A in Supplementary Material, PSDM and SDM-SC did not show any alterations of proliferative capability. However, a higher number of cells in S phase were observed in PSDM-SCs compared to SDM-SCs (28 vs. 16% after 24 h and 19 vs. 9% after 48 h). In addition, cell viability revealed marginal differences among the analyzed samples and the minimal viability rate was observed in SDM-SCs at 24 h post treatment (approximately 93%, Figure S2B in Supplementary Material).

The ability to fuse under stress condition was evaluated by immunostaining. The H_2_O_2_ treatment was sufficient to dramatically reduce myogenic differentiation in SDM-SCs while PSDM-SCs were not affected. This suggests that, differently from SDM-SCs, PSDM-SCs are resistant to oxidative stress.

### Resting cytoplasmic [Ca^2+^]_i_

Myogenesis is a strictly Ca^2+^-dependent process and according to Bijlenga et al. (2000) the biophysical properties of specific ionic channels are important actors in the fusion process. We measured the resting [Ca^2+^]_i_ in myoblasts derived from somitic and presomitic, young, old, and GRMD muscles. The findings for PSDM GRMD SCs showed an increasing resting intracellular calcium concentration (127.0 ± 18 nM, *n* = 24) as compared to the somitic ones (SDM GRMD SCs; 63.5 ± 10 nM, *n* = 50) (Figure 3D). On the contrary, SDM GRMD and SDM WT young had similar content in cytoplasmic calcium at resting condition (63.5 ± 10 nM, *n* = 24; 65.0 ± 6 nM, *n* = 27) (Figure 3D). Likewise, the old SDM-SCs presented a higher level of resting [Ca^2+^]_i_ as compared to young and GRDM, but differently from those that did not show an increase of Ca^2+^ level in PSDM.

### Ca^2+^ transient induced by extracellular stimuli

To verify the presence of functional receptors, Ca^2+^ imaging experiments were performed on single myoblasts isolated from somitic and presomitic muscles, young, old, and GRMD. The physiological agents used on living cells were: (1) 100 μM ATP, which mainly causes a release of Ca^2+^ via IP_3_/PKC (protein kinase) pathway in muscle cells; (2) 400 μM nicotine, which acts on nicotine receptors, a typical channel of neuromuscular junction; (3) 40 mM KCl, which induces a chemical depolarization of external membranes on myotube.

As shown in Table 3, all samples analyzed were responsive to 100 μM ATP with no significant differences, demonstrating that the release of calcium via IP_3_ pathway was unaffected by aging, or disease, or embryonic origin of SCs.

**Table 3:** **Small molecule responsiveness of undifferentiated SCs; values are expressed as a percentage**.

	Young		Old		GRMD	
	SDM	PSDM	SDM	PSDM	SDM	PSDM
Nicotine	0	90	3	80	50	65
KCl	6	0	3	35	4	66
ATP	100	65	93	65	100	56

Surprisingly, a high percentage of stimulated cells of young (90%), old (80%), and GRMD (65%) from PSDM were responsive to 400 μM nicotine, revealing the presence of cholinergic receptors on the external membrane of undifferentiated cells. Furthermore, PSDM-SCs from old (35%) and GRDM (65.0%) dogs were also shown to respond to depolarizing stimulus (40 mM KCl).

### Muscle-specific miRNAs in WT canine SCs

**Figure 4:**
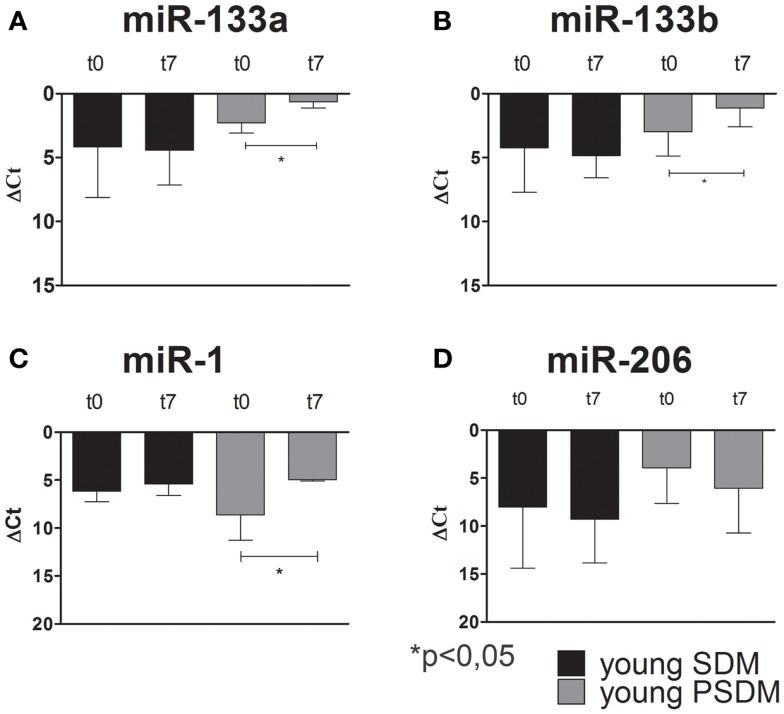
**Relative miR-133a, miR-133b, miR-1, and miR-206, expression in canine SCs measured by quantitative real-time PCR**. SCs were isolated from SDM and PSDM of young dogs. Note that miR-133a **(A)** and miR-133b **(B)** are upregulated only in PSDM-SCs after 7 days of differentiation (t7). miR-1 is upregulated only in PSDM-SCs **(C)** after 7 days of differentiation. At variance, miR-206 is highly expressed in PSDM-SCs during proliferation **(D)**. The ubiquitously produced miR-16 was used as an internal control. Three independent experiments were performed in triplicates and statistically analyzed using ANOVA. Data are presented as mean ± SD; *p < 0.05.

**Figure 5:**
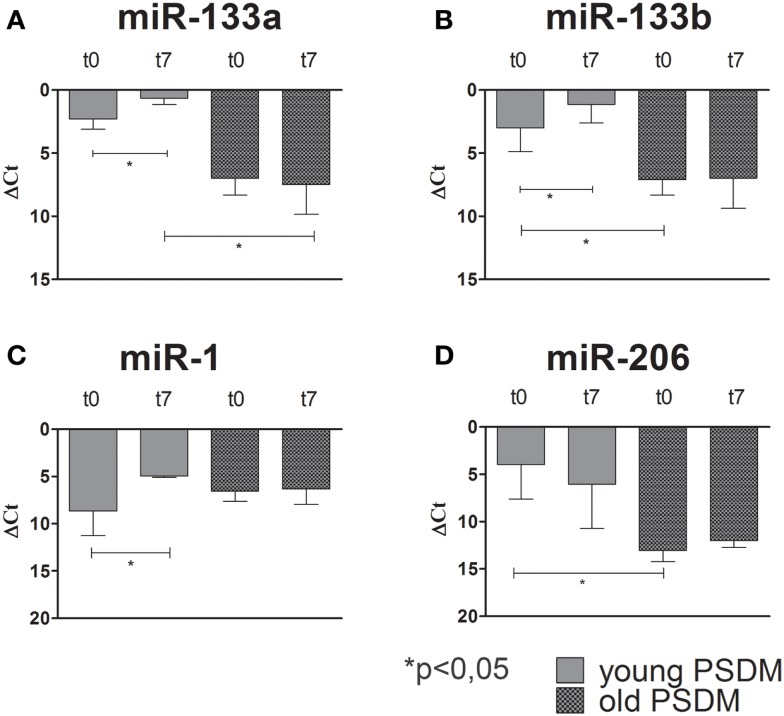
**Relative miR-133a, miR-133b, miR-1, and miR-206, expression in old canine PSDM-SCs measured by quantitative real-time PCR**. In old PSDM-SCs, miR-133a **(A)** and miR-133b **(B)** are downregulated in both proliferating and differentiating cells. miR-1 and miR-206 regulations seem age-dependent, since old PSDM-SCs did not upregulate miR-1 during differentiation **(C)**, while miR-206 is not highly expressed during proliferation **(D)**. The ubiquitously produced miR-16 was used as an internal control. Three independent experiments were performed in triplicates and statistically analyzed using ANOVA; Data are presented as mean ± SD; *p < 0.05.

**Figure 6:**
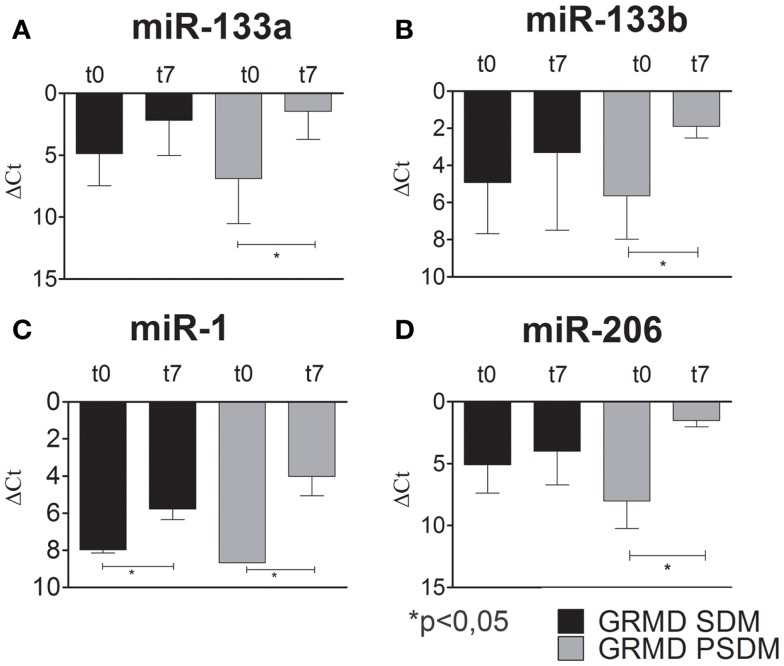
**Relative miR-133a, miR-133b, miR-1, and miR-206 expression in GRMD SCs measured by quantitative real-time PCR**. SCs were isolated from SDM and PSDM of GRMD dogs. Consistent with data obtained with WT canine SCs, miR-133a and miR-133b are highly expressed after 7 days of differentiation in PSDM-SCs. At variance, miR-133a and miR-133b are upregulated in SDM-SCs after 7 days of differentiation **(A,B)**. Intriguingly, miR-1 and miR-206 are highly upregulated after 7 days of differentiation in PSDM-SCs **(C,D)**. Three independent experiments were performed in triplicates and statistically analyzed using ANOVA; miR-16 was used as an internal control. Data are presented as mean ± SD; *p < 0.05.

Some miRNAs are involved in post-transcriptional regulation of gene expression during skeletal muscle development. Therefore, we examined the expression of miR-1, miR-206, miR-133a, miR-133b (Figures 4–6), collectively known as myo-miRNAs and using miR-16 ubiquitously expressed as internal control. The results reported in Figures 4A,B showed no differences in miR-133a and miR-133b expression after 7 days of differentiation (t7) in isolated SCs from young SDM. However, we found that miR-133a and miR-133b were highly expressed in PSDM-SCs upon differentiation. The myogenic miR-1 was slightly upregulated at t7 only in PSDM-SCs from young donors (Figure 4C). miR-206 was similarly expressed in young PSDM- and to SDM-SCs (Figure 4D). With aging, the expression levels of miR-133a (Figure 5A) and miR-133b (Figure 5B) resulted affected and old PSDM-SCs did not positively regulate those miRNAs, whereas the expression of miR-1 was not modified in PSDM-SCs from old dogs (Figure 5C). Also, miR-206 in PSDM-SCs from old donors was found downregulated as compared to values from young donors and not modified during myogenic differentiation (Figure 5D). In summary, the expressions of myomiRs appeared to be modulated in SCs isolated from PSDM and this phenomenon seems to be age-dependent.

### Muscle-specific miRNAs in GRMD SCs

Recent studies have shown that changes in miRNA expressions are associated with various skeletal muscle disorders, including muscular dystrophy (Eisenberg et al., 2009; Williams et al., 2009). However, the contradictory literature motivated the investigation for the expression profile of myomiRs during myogenic differentiation of dystrophic SCs. In fact, some authors referred to myo-miRNAs as markers of muscle regeneration while others support their critical role to sustain muscle degeneration. As for WT SCs, we studied the expression of myogenic miRNAs in SCs isolated from SDM and PSDM of GRMD dogs. As shown in Figure 6, miR-133a and miR-133b were upregulated at day 7 in SCs isolated from GRMD PSDM (Figures 6A,B). This result was consistent with WT expression. In addition, similarly to WT cells, miR-133a and miR-133b were not upregulated in SDM-SCs upon differentiation (Figures 6A,B). Likewise, miR-1 and miR-206 were markedly upregulated in PSDM-SCs after 7 days of differentiation in GRMD dogs (Figures 6C,D) and not in GRDM SDM-SCs (Figures 6C,D). The expression profile of myomiRs appeared to be affected by the dystrophic niche and miR-206 miR-1 seem to be primarily involved since they are highly expressed in differentiated SCs from both SDM and PSDM of GRMD samples.

## Discussion

In the last decades, SCs have been shown as muscle stem cells responsible for tissue regeneration in adulthood (Scharner and Zammit, 2011). Most of clinically relevant protocols for testing potential therapeutic approaches have been performed in small animal models, meaning mice and rats (Blau, 2008; Kuang and Rudnicki, 2008). However, large animal models have been considered essential to better mimic human diseases, to validate outcome measures, and to obtain reliable protocols for novel pharmaceutical, gene, and cell therapies. Thus, GRMD dogs represent a valuable large animal model to perform preclinical studies (Kornegay et al., 1988; Sharp et al., 1992). However, our knowledge on biological behavior of muscle regeneration in canine tissues remains primitive and requires further investigations.

In this work, extensive analysis for the biological properties of WT (young and old) and GRMD SCs isolated from somitic (SDM) and presomitic (PSDM) muscles are carried out. Although the gross morphology is quite similar in these muscles, several studies revealed unexpected molecular and cellular differences depicting peculiar biological characteristics in proliferation, lifespan, and differentiation potential of SDM and PSDM-SCs (Ono et al., 2010). With enzymatic dissociation it was possible to isolate SCs in all biopsies obtained by different research centers including Ecole de Veterinaire Maison-Alfort, Veterinary and University Clinics of Padua. In general, there were no observed differences in terms of SC quality, among the samples obtained by fresh or frozen biopsies. The proliferative lifespans of SCs derived from SDM and PSDM in both WT and GRMD dogs at different ages was also considered and compared. While the proliferative ability of SCs isolated from young SDM was approximately 20 population doublings before replicative senescence, SCs from young PSDM had 25 divisions *in vitro*. These differences were remarkable in WT old muscle as SCs isolated from PSDM stop to proliferate after 28 divisions while the cells derived from SDM had only 18 divisions. At later stage although SDM-SCs isolated from GRMD biopsies were phenotypically indistinguishable from WT SCs in *ex vivo* cultures, the cells showed a slower proliferative rate compared to the young WT SCs. Indeed, after 40 days they reached about 18 divisions compared to 30 days necessary to SDM-SCs isolated from WT samples. These findings suggest that SDM-SCs in GRMD dogs were exhausted by multiple attempts to regenerate lost tissues resulting in a limited proliferative ability. Nevertheless, they were prone to differentiate into myotubes much faster when compared to their WT counterparts. It is possible that when cultured *in vitro* they recapitulate their *in vivo* needs, i.e., to faster replace damaged myofibers. In this view, several articles recently showed a potential cell memory in reprogrammed cells (Kim et al., 2010; Polo et al., 2010; Quattrocelli et al., 2011). Our group also previously proposed that the SC pool is affected by the same lifestyle or pathological condition of the muscle in which they reside (Fulle et al., 2004). Similarly, SCs generated from degeneration/regeneration cycles appear to adapt to their biological behavior to better counteract the following degenerative processes (La Rovere et al., unpublished observation).

After isolation SCs are characterized by a period of rapid growth upon serial passages, until their proliferation rate gradually slows down and ultimately enters a non-dividing state called replicative senescence (Allsopp et al., 1995). Each cell division is characterized by a decrease of telomerase length and that has been proposed as a mitotic clock that regulates proliferative capacity of somatic cells *in vitro* (Di Donna et al., 2000). Thus, the higher telomerase activity observed in PSDM-SCs supports the hypothesis that those cells preserve a better proliferation capability compared to SDM-SCs, and consistently their lifespan is prolonged.

In our study, desmin expression was used as internal standard for normalization to avoid misinterpretation of comparative evaluations. Generally, low content of myogenic cells were obtained from head muscles compared to somite muscles and this difference was independent of sample age and between WT and GRMD. The FI and skeletal muscle differentiation potential of WT and GRMD SCs was evaluated and analyzed. Our data show that WT limb muscles (SDM) contained a higher percentage of activated SCs compared to PSDM, as indicated by Pax7 and MyoD expression analysis. Conversely, SDM-SCs from GRMD dogs showed a lower percentage of Pax7 and MyoD positive cells, whereas craniofacial muscle (PSDM) contained significantly higher numbers of Pax7 and MyoD positive SCs. Overall, our analyses strongly support the idea that PSDM-SCs retain a higher myogenic potential compared to the SDM-SCs isolated from GRMD muscles. Despite a higher number of MyoD positive cells in PSDM-SCs isolated from GRDM dogs, they show less differentiation capacity compared to the WT counterparts.

To verify if PSDM-SCs have an intrinsic capability to resist to toxic insults, or whether their invulnerable behavior can be justified from specific niche characteristics, the response of both PSDM-SCs and SDM-SCs to hydrogen peroxide treatment has been assessed. Notwithstanding that the proliferation ability of SCs from PSDM and SDM samples were not affected, 100 nM H_2_O_2_ treatment impaired the myogenic differentiation exclusively in SDM-SCs.

To fully investigate if this reduced differentiative capability was due to high oxidant levels, ROS contents have been evaluated. Although for a long time the ROS formation has been believed harmful, evidences are accumulating showing the key role of ROS presence and production in cell signaling. In addition, changes in ROS content seem to be modulated during myogenic processes (Musarò et al., 2010). It was previously observed that human SC derived from old subjects showed to have a high oxidative stress with respect to young samples (Fulle et al., 2004; Beccafico et al., 2007; Pietrangelo et al., 2009). This was associated to higher [Ca^2+^]_i_, probably due to alteration of calcium homeostasis regulatory proteins functionality. Here, it is confirmed that elderly SDM and PSDM canine SCs showed high ROS levels, however, dystrophic SCs displayed ROS levels similarly to young WT samples. In all PSDM-SCs, high responsiveness to nicotine determined the characteristic calcium transients and revealed the presence of nicotinic receptors on undifferentiated cells (Krause et al., 1995). SCs express low levels of functional nicotinic acetylcholine receptors (nAChR) before myosin becomes detectable (Grassi et al., 2004) and considered to be an early marker of myogenic differentiation. Thus, this opens up new cues to examine presomitic aged and dystrophic SCs that showed to still remain functional and able to fuse with existing fibers.

To date, the crucial miRNAs involved in skeletal muscle differentiation are miR-1, miR-206, miR-133a, miR-133b and are collectively known as myomiRs. miR-1 and miR-206 are key myo-miRNAs for Mef2c-dependent terminal myogenic differentiation. miR-1 and miR-206 act as positive controls for the progression of differentiation via Notch 3 and utrophin inhibition (Rosenberg et al., 2006; Gagan et al., 2012). Several studies have shown that changes in miRNA expressions are associated with various skeletal muscle disorders, including muscular dystrophy (Eisenberg et al., 2009; Williams et al., 2009). Thus, we examined the expression of myomiRs during proliferation (day 0) and differentiation (day 7) of SCs isolated from WT and GRMD samples. Our data showed that miR-133a and miR-133b, involved in early myogenic differentiation, were upregulated after 7 days of differentiation in WT PSDM. However, this upregulation disappeared with aging and the PSDM-SCs acted similarly to SDM-SCs. miR-1 is also upregulated at day 7 in WT PSDM-SCs and again aging affected negatively its modulation. miR-206 is highly expressed in young WT PSDM samples while in aged samples it is much less expressed. Overall from the collected results, myomiRs are clearly modulated in PSDM-SCs of WT canine muscles and this regulation is lost with aging. In the GRMD, a similar modulation for miR-133a, miR-133b, and miR-1 was found in the PSDM-SCs. In addition, miR-206 was strongly upregulated at day 7 of differentiation. Intriguingly, myomiR upregulation was observed in SDM-SCs of GRMD dogs that feature worse locomotion ability. In this view, myomiR overexpression could sustain the muscle chronic degeneration of GRMD dogs by affecting the expression of myomiR target genes.

Emerging literature confirmed that the expression of miR-206 is essentially confined to skeletal muscle (Baskerville and Bartel, 2005; Liang et al., 2007) and involved in muscle differentiation by repressing the expression of DNA polymerase A, connexin 43, follistatin-like 1, and utrophin. Intriguingly, miR-206 was found highly expressed in the diaphragm of *mdx* mouse, the murine counterpart of GRMD dogs, and not in the hindlimb muscles (McCarthy et al., 2007). This result is significant because the diaphragm of the *mdx* mouse displays DMD phenotype differently from hindlimb muscles. McCarthy et al. (2007) proposed that increased miR-206 expression may contribute to the chronic pathology of *mdx* diaphragm. In fact, miR-206 represses the expression of genes, including utrophin, that otherwise would serve as compensatory function. Utrophin is indeed a verified target of miR-206, and this observation explains at least in part the higher expression of utrophin protein in the *mdx* hindlimb musculature where miR-206 is barely detectable. Conversely, Eisenberg et al. (2007) showed recently the expression profiling of 10 different human dystrophies, including DMD, in which miR-206 expression was similar to one observed in control muscles. One possible explanation for this discrepancy is again the different muscles used to evaluate miRNA expressions. In conclusion, the expression profile of MRFs and myomiRs reveals a unique molecular signature in canine SCs. In addition, miR-206 seems to be primarily involved in GRMD SC impairment, although its precise role needs to be carefully considered in the light of discordant literature.

## Conflict of Interest Statement

The authors declare that the research was conducted in the absence of any commercial or financial relationships that could be construed as a potential conflict of interest.

## Supplementary Material

The Supplementary Material for this article can be found online at http://www.frontiersin.org/Journal/10.3389/fnagi.2014.00090/abstract

Figure S1 **Immunofluorescence analysis for Ki67 expression in early and late passages of SC cultures**. Examples of Ki67 staining indicating that up to 30% of SCs are proliferating in both SDM and PSDM samples. Consistent with growth curve data at late stage (30 days) while up to 10% of PSDM-SCs (22 PDL) are still positive for Ki67, SDM-SCs (20 PDL) stopped proliferation and the majority of cells present large nuclei (red arrow heads). Ki67 positive nuclei (green arrow heads) are much smaller compared to negative ones (right lower panel). Bar = 100 μm.

Figure S2 **Effect of hydrogen peroxide on SC differentiation**. SCs were isolated from SDM and PSDM of WT dogs and harvested after 24 and 48 h of treatment with 100 nM hydrogen peroxide. SCs did not show any differences under this condition. However, a higher number of cells in S phase were observed in PSDM-SCs compared to SDM-SCs (28 vs. 16% after 24 h and 19 vs. 9% after 48 h) **(A)**. Cell viability was assessed using Countess Automated Cell Counter (Life Technologies) after trypan blue staining **(B)**. The H_2_O_2_ treatment was sufficient to dramatically reduce myogenic differentiation in SDM-SCs while PSDM-SCs were not affected **(C)**. Bar = 100 μm.
