# Supplementary figures and images for: Myogenic Potential of Canine Craniofacial Satellite Cells
Source: Front Aging Neurosci. 2014 May 13;6:90. doi: 10.3389/fnagi.2014.00090 (PMC4026742; doi:10.3389/fnagi.2014.00090)

SDM

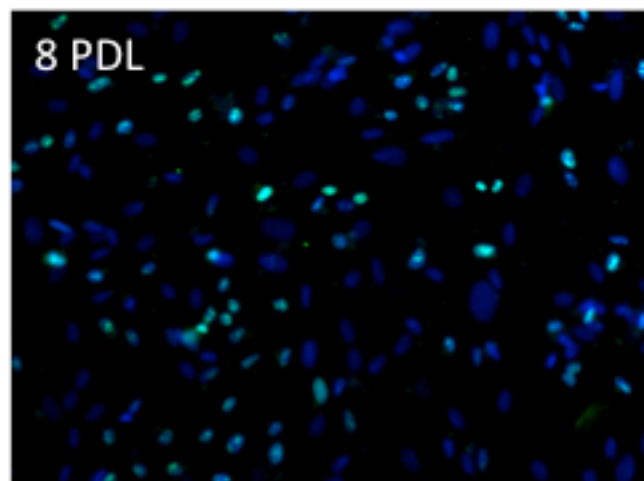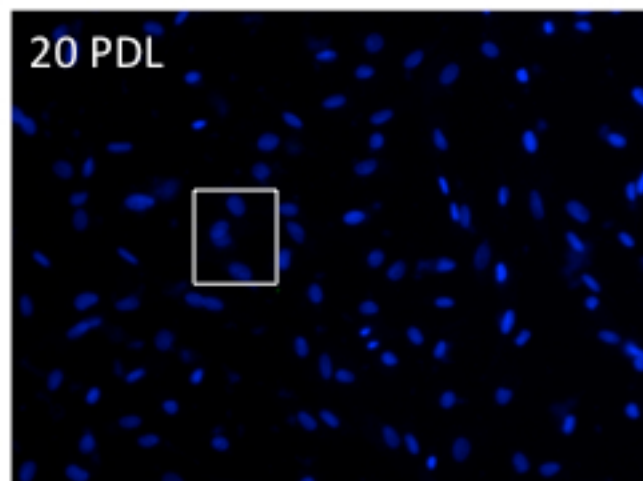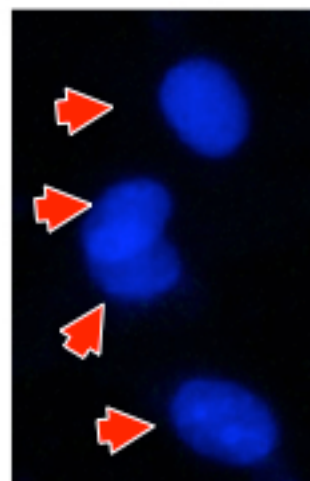

PSDM

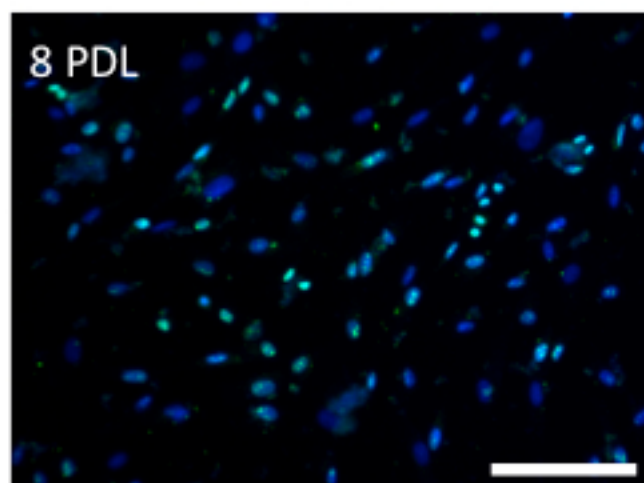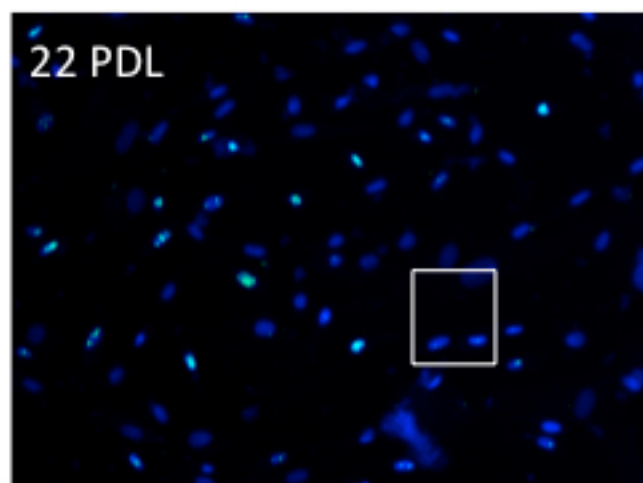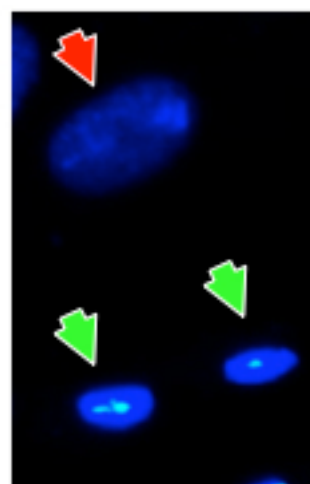

Day 15

DAY 30

Supplement: Figure S1 — Immunofluorescence analysis for Ki67 expression in early and late passages of SC cultures. Examples of Ki67 staining indicating that up to 30% of SCs are proliferating in both SDM and PSDM samples. Consistent with growth curve data at late stage (30 days) while up to 10% of PSDM-SCs (22 PDL) are still positive for Ki67, SDM-SCs (20 PDL) stopped proliferation and the majority of cells present large nuclei (red arrow heads). Ki67 positive nuclei (green arrow heads) are much smaller compared to negative ones (right lower panel). Bar = 100 μm. [file Presentation1.ZIP › Figure S1.pdf]

A

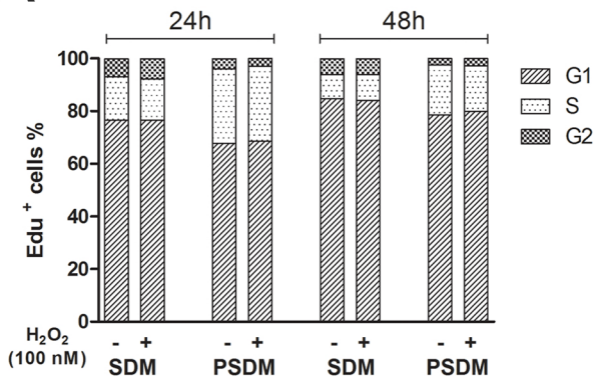

B

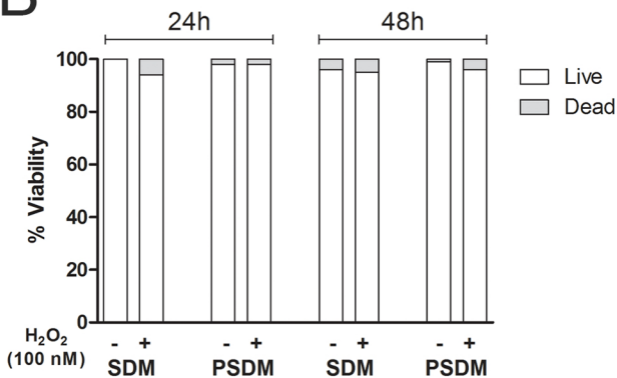

C

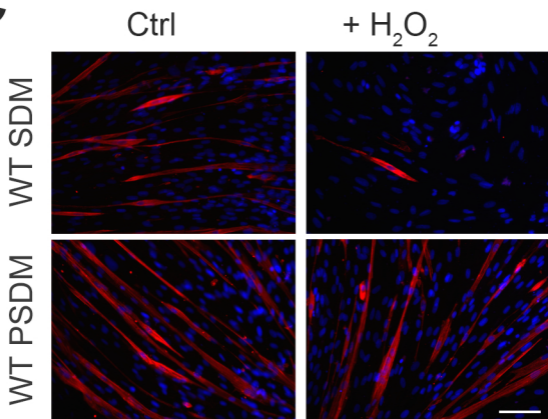

Supplement: Figure S1 — Immunofluorescence analysis for Ki67 expression in early and late passages of SC cultures. Examples of Ki67 staining indicating that up to 30% of SCs are proliferating in both SDM and PSDM samples. Consistent with growth curve data at late stage (30 days) while up to 10% of PSDM-SCs (22 PDL) are still positive for Ki67, SDM-SCs (20 PDL) stopped proliferation and the majority of cells present large nuclei (red arrow heads). Ki67 positive nuclei (green arrow heads) are much smaller compared to negative ones (right lower panel). Bar = 100 μm. [file Presentation1.ZIP › Figure S2.pdf]
